# Exercise‐induced appetite suppression: An update on potential mechanisms

**DOI:** 10.14814/phy2.70022

**Published:** 2024-08-26

**Authors:** Seth F. McCarthy, Jessica A. L. Tucker, Tom J. Hazell

**Affiliations:** ^1^ Department of Kinesiology and Physical Education Wilfrid Laurier University Waterloo Ontario Canada

**Keywords:** anorexigenic, appetite regulation, energy intake, fullness, gut hormones, hunger, orexigenic, satiety

## Abstract

The first systematic reviews of the effects of exercise on appetite‐regulation and energy intake demonstrated changes in appetite‐regulating hormones consistent with appetite suppression and decreases in subsequent relative energy intake over a decade ago. More recently, an intensity‐dependent effect and several potential mechanisms were proposed, and this review aims to highlight advances in this field. While exercise‐induced appetite suppression clearly involves acylated ghrelin, glucagon‐like peptide‐1 may also be involved, though recent evidence suggests peptide tyrosine tyrosine may not be relevant. Changes in subjective appetite perceptions and energy intake continue to be equivocal, though these results are likely due to small sample sizes and methodological inconsistencies. Of the proposed mechanisms responsible for exercise‐induced appetite suppression, lactate has garnered the most support through in vitro and in vivo rodent studies as well as a growing amount of work in humans. Other potential modulators of exercise‐induced appetite suppression may include sex hormones, growth‐differentiation factor 15, Lac‐Phe, brain‐derived neurotrophic factor, and asprosin. Research should focus on the mechanisms responsible for the changes and consider these other modulators (i.e., myokines/exerkines) of appetite to improve our understanding of the role of exercise on appetite regulation.

## 1 INTRODUCTION

**FIGURE 1:**
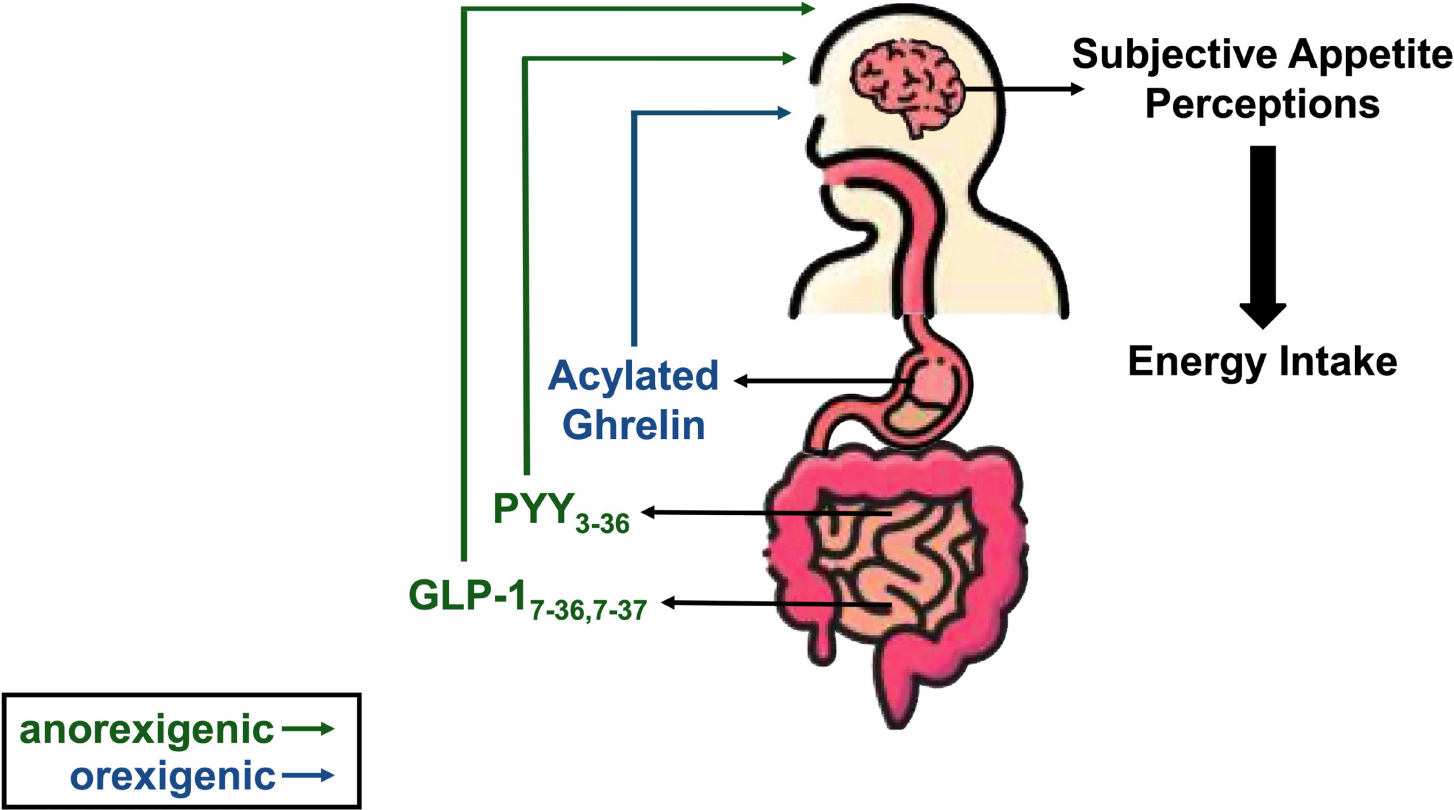
Brief overview of the physiological regulation of appetite. GLP‐1, glucagon‐like peptide‐1; PYY, peptide tyrosine tyrosine.

Over the past three decades, a host of research studies have assessed the effect of acute exercise on appetite regulation (Stensel, 2010). While a detailed description of the control of appetite is beyond the scope of this review and previously well explained (Murphy & Bloom, 2006), a brief overview is summarized in Figure 1. The regulation of energy intake is a complex system involving behavioral, environmental, and physiological factors (King et al., 2012) however, this review will focus on the physiological. On a meal‐to‐meal basis, the physiological regulation of energy intake involves fluctuations in orexigenic (appetite‐stimulating) and anorexigenic (appetite‐suppressing) peripheral appetite hormones (Cummings & Overduin, 2007). Many of these hormones circulate in active and inactive forms, where the active forms such as acylated ghrelin, glucagon like peptide_7‐36/7–37_ (GLP‐1), and peptide tyrosine tyrosine_3‐36_ (PYY) are more important in appetite regulation (Chelikani et al., 2005; Orskov et al., 1993; Yang et al., 2008). Changes in these hormones can alter perceptions of appetite by acting on specific brain regions (hypothalamus/brainstem) altering the release of neuropeptides responsible for changes in feeding behavior (Harrold et al., 2012; Parker & Bloom, 2012).

Several reviews (Hazell et al., 2016; Schubert et al., 2013, 2014) were pivotal in summarizing the relationship between acute exercise and appetite. A decade ago two meta‐analyses demonstrated energy intake is not increased postexercise to compensate for exercise energy expenditure (Schubert et al., 2013) and these changes involve decreases in acylated ghrelin as well as increases in PYY and GLP‐1 (Schubert et al., 2014). While other peripheral hormones (e.g., pancreatic polypeptide, leptin, cholecystokinin) are involved in the regulation of appetite, the previous meta‐analysis focused on acylated ghrelin, PYY, and GLP‐1 as these are most often assessed with exercise. The influential research included in these two meta‐analyses focused on the postexercise responses, and a subsequent review highlighted an intensity‐dependent role of exercise as well as proposed several physiologically plausible mechanisms unique to higher intensity exercise that may be responsible for the changes in appetite‐regulating hormones (Hazell et al., 2016). These reviews (Hazell et al., 2016; Schubert et al., 2013, 2014) highlighted that the duration of exercise does not appear to be important and duration has received little attention since and will not be discussed. This review will update these key exercise and appetite reviews published nearly a decade ago (Hazell et al., 2016; Schubert et al., 2013, 2014) and highlight current advances and future directions.

## 2 THE APPETITE RESPONSE TO EXERCISE

**FIGURE 2:**
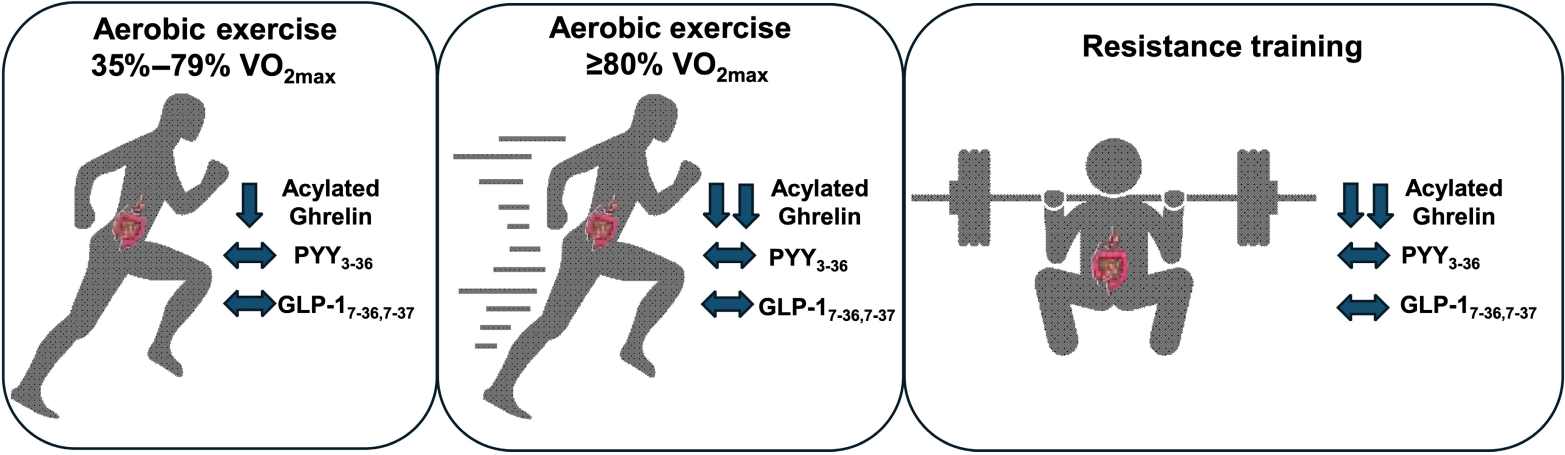
Overview of the effect of exercise intensity and mode on key appetite‐regulating hormones. GLP‐1, glucagon‐like peptide‐1; PYY, peptide tyrosine tyrosine.

Acute bouts of exercise (aerobic and resistance) can create an energy deficit (Schubert et al., 2013) involving changes in key appetite‐regulating hormones (Schubert et al., 2014). While a necessary first step in summarizing evidence regarding exercise and appetite, these reviews did not include exercise intensities above 80% maximal oxygen consumption (V̇O_2max_), characterized as vigorous‐intensity continuous training, high‐intensity interval training (HIIT), or sprint‐interval training. A thorough description of these protocols is available elsewhere (Coates et al., 2023). Additionally, both total and active forms of the appetite‐regulating hormones were included and research focusing on the active forms which are more physiologically relevant, will be necessary to continue improving our understanding of the effects of exercise on appetite regulation (Chelikani et al., 2005; Orskov et al., 1993; Yang et al., 2008). Therefore, this section aims to summarize recent developments over the last decade that have improved our understanding of how exercise alters appetite (Figure 2).

### 2.1 Aerobic exercise

The previous meta‐analysis reported exercise had a small effect (Cohen's *d* = −0.20) suppressing acylated ghrelin concentrations (Schubert et al., 2014) where ~45% of the included studies demonstrated an acute bout of moderate‐intensity continuous training (MICT; 30–90 min at ~60%–72% VO_2max_) suppressed acylated ghrelin postexercise ~9%–56% (Beer et al., 2020; Broom et al., 2007, 2009; Deighton et al., 2013; King, Miyashita, et al., 2010; Shorten et al., 2009; Vatansever‐Ozen et al., 2011; Wasse, Sunderland, King, Batterham, & Stensel, 2012; Wasse, Sunderland, King, Miyashita, & Stensel, 2012). While the majority of papers included young recreationally‐active males (Alajmi et al., 2016; Balaguera‐Cortes et al., 2011; Becker et al., 2012; Beer et al., 2020; Bornath et al., 2023; Broom et al., 2007, 2017; Deighton et al., 2013, 2014; Douglas et al., 2017; Finlayson et al., 2009; Hagobian et al., 2013; Hazell et al., 2017; Holliday & Blannin, 2017; Imbeault et al., 1997; Islam et al., 2017; Kelly et al., 2012; King et al., 1994, 1997, 2017; King, Miyashita, et al., 2010; King, Wasse, et al., 2010; King, Wasse, Ewens, et al., 2011; Martins et al., 2007; McCarthy et al., 2023; Metcalfe et al., 2015; Panissa et al., 2016; Shorten et al., 2009; Ueda, Yoshikawa, Katsura, Usui, Nakao, & Fujimoto, 2009; Vanderheyden et al., 2020; Vatansever‐Ozen et al., 2011; Wasse, Sunderland, King, Batterham, & Stensel, 2012; Wasse, Sunderland, King, Miyashita, & Stensel, 2012), there were some studies that included young or middle‐aged females (Alajmi et al., 2016; Beer et al., 2020; Douglas et al., 2017; Finlayson et al., 2009; Hagobian et al., 2013; Halliday et al., 2021; Hallworth et al., 2017; Hazell et al., 2017; Howe et al., 2016; Kamemoto et al., 2022; King et al., 1996; Larson‐Meyer et al., 2012; Martins et al., 2007, 2015; McCarthy et al., 2023; Moniz et al., 2023), middle‐aged men (McCarthy et al., 2023), as well as individuals experiencing overweight/obesity (Bornath et al., 2023; Hagobian et al., 2009; Holmstrup et al., 2013; Hopkins et al., 2014; Larsen et al., 2017; Metcalfe et al., 2015; Sim et al., 2014; Tobin et al., 2021; Unick et al., 2010). About half the studies demonstrated a nonsignificant change in acylated ghrelin with values ranging from ~1% to 20% (Balaguera‐Cortes et al., 2011; Hagobian et al., 2013; King, Wasse, et al., 2010; King, Wasse, Ewens, et al., 2011; King, Wasse, & Stensel, 2011; Larsen et al., 2019a; McCarthy et al., 2023; Panissa et al., 2016; Sim et al., 2014; Unick et al., 2010) and one study demonstrated an ~13% increase (Larson‐Meyer et al., 2012) though we cannot rationalize this. Research published after this review aligns, as most demonstrate (~85%) decreases in acylated ghrelin following MICT (~3%–46%) (Alajmi et al., 2016; Bornath et al., 2023; Broom et al., 2017; Howe et al., 2016; King et al., 2017; Larsen et al., 2017). Some work continues to measure total ghrelin, and the majority of results suggest no change (~2%–11%) (Douglas et al., 2017; Martins et al., 2007; Ouerghi et al., 2019; Tobin et al., 2021; Ueda, Yoshikawa, Katsura, Usui, Nakao, & Fujimoto, 2009), though one demonstrates a decrease (~22%) (Kelly et al., 2012). Taken together, 15 of 27 studies demonstrate an exercise‐induced suppression of acylated ghrelin. In addition, higher intensity exercise (>80% V̇O_2max_) elicit similar magnitude reductions (~24%–65%) (Beer et al., 2020; Deighton et al., 2013); Broom et al., 2017; Islam et al., 2017; McCarthy et al., 2023; Panissa et al., 2016; Sim et al., 2014; Beaulieu et al., 2015; Larsen et al., 2019b but consistent responses as all studies employing VICT, HIIT, or SIT suppress acylated ghrelin (Beaulieu et al., 2015; Beer et al., 2020; Broom et al., 2017; Deighton et al., 2013; Islam et al., 2017; Larsen et al., 2019b; McCarthy et al., 2023; Panissa et al., 2016; Sim et al., 2014) with several key studies supporting an intensity‐dependent response (Islam et al., 2017; McCarthy et al., 2023; Panissa et al., 2016; Sim et al., 2014).

Regarding PYY, the previous meta‐analysis reported exercise had a small effect (Cohen's *d* = 0.24) increasing PYY (Schubert et al., 2014). Studies included in the meta‐analysis demonstrated a ~4%–25% increase in active (Larson‐Meyer et al., 2012) and total PYY (Broom et al., 2009; Deighton et al., 2013; Ueda, Yoshikawa, Katsura, Usui, & Fujimoto, 2009; Wasse, Sunderland, King, Batterham, & Stensel, 2012) or no change in total PYY (~1%–9%) (Balaguera‐Cortes et al., 2011; Kelly et al., 2012; Martins et al., 2007; Shorten et al., 2009) following an acute MICT session. Research conducted after the meta‐analysis (Schubert et al., 2014) continues to demonstrate the total PYY response is equivocal as several demonstrate increases (~6%–30%) (Afrasyabi et al., 2019; Douglas et al., 2017; Islam et al., 2017) or no change in total PYY (~3%–13%) (Halliday et al., 2021; Holmstrup et al., 2013; Tobin et al., 2021). Recent studies assessing active PYY demonstrate no change (~4%–7%) (Bornath et al., 2023; McCarthy et al., 2023; Moniz et al., 2023) suggesting active PYY is not responsive to exercise. Exercise‐intensity (i.e., VICT, HIIT, or SIT protocols) does not appear to influence the response for active (Howe et al., 2016; Martins et al., 2015; McCarthy et al., 2023; Panissa et al., 2016) or total PYY (Hallworth et al., 2017; Hazell et al., 2017; Islam et al., 2017; Metcalfe et al., 2015; Sim et al., 2014) suggesting PYY may not be relevant for exercise‐induced appetite suppression.

The previous meta‐analysis reported that exercise had a small effect (Cohen's *d* = 0.28), increasing GLP‐1 concentrations (Schubert et al., 2014). Studies included in the meta‐analysis demonstrated increases in active (33%) (Ueda, Yoshikawa, Katsura, Usui, Nakao, & Fujimoto, 2009) and total GLP‐1 (18%–38%) (Larson‐Meyer et al., 2012; Martins et al., 2007; Ueda, Yoshikawa, Katsura, Usui, & Fujimoto, 2009) following an acute MICT session with one study demonstrating an 8% decrease in total GLP‐1 (Unick et al., 2010). Despite being included in the meta‐analysis, there were only 5 studies available and much more research has been conducted in recent years. This more recent work is equivocal where several studies demonstrate increased active (~24%) (Islam et al., 2017) or total GLP‐1 (~15%–97%) (Douglas et al., 2017; Hallworth et al., 2017; Hazell et al., 2017; Howe et al., 2016) and several show no change (~5%–16%) in active (Bornath et al., 2023; McCarthy et al., 2023) or total GLP‐1 (Halliday et al., 2021; Tobin et al., 2021) following acute MICT sessions. Overall, 7 of 10 studies demonstrate increases in total GLP‐1 postexercise though only 2 of 4 studies demonstrate increases in active GLP‐1. Similar to PYY, exercise intensity does not influence active (Islam et al., 2017) or total GLP‐1 (Hallworth et al., 2017; Hazell et al., 2017; Howe et al., 2016) responses and more work is required to determine if changes in GLP‐1 are relevant for exercise‐induced appetite suppression.

The effect of acute exercise on subjective appetite perceptions has not been systematically assessed. Studies completed prior to 2014 (consistent with meta‐analyses) are equivocal, demonstrating statistically significant decreases (~9%–49%) (Broom et al., 2007, 2009; Holmstrup et al., 2013; King et al., 1994; King, Miyashita, et al., 2010; Ueda, Yoshikawa, Katsura, Usui, & Fujimoto, 2009; Unick et al., 2010) or nonsignificant reductions (~3%–21%) (King et al., 1996, 1997; King, Wasse, et al., 2010; King, Wasse, Ewens, et al., 2011; King, Wasse, & Stensel, 2011; Martins et al., 2007) following an acute MICT sessions. More recent studies measuring overall subjective appetite (using a cumulative score from 4 individual questions) (Flint et al., 2000) following MICT have demonstrated similar results with statistically significant decreases (~19%–57%) (Broom et al., 2017; Hallworth et al., 2017; Hazell et al., 2017; Islam et al., 2017; Larsen et al., 2019a; Panissa et al., 2016) or nonsignificant reductions (~1%–24%) (Bornath et al., 2023; Douglas et al., 2017; Halliday et al., 2021; Martins et al., 2015; McCarthy et al., 2023; Metcalfe et al., 2015; Sim et al., 2014). Several of the studies demonstrating nonsignificant differences demonstrate comparable magnitudes of change to the statistically significant changes, highlighting the potential for type II error in research with small sample sizes. Additionally, studies that have compared exercise intensities show equivocal results with some studies identifying an intensity‐dependent effect (Deighton et al., 2013; Hallworth et al., 2017; Islam et al., 2017) though most find no differences (Broom et al., 2017; Hazell et al., 2017; Larsen et al., 2019a; McCarthy et al., 2023; Metcalfe et al., 2015; Panissa et al., 2016; Sim et al., 2014; Ueda, Yoshikawa, Katsura, Usui, & Fujimoto, 2009). It is important to note most exercise and appetite‐related research is powered to detect differences in appetite‐regulating hormones only, and differences in how subjective appetite is measured makes magnitudes of change difficult to compare.

Changes in appetite hormones and perceptions are important to assess, though ultimately the change in behavior (energy intake) are crucial. The previous meta‐analysis reported energy intake is not increased postexercise to compensate for the during‐exercise energy expenditure (Schubert et al., 2013). The studies in the meta‐analysis demonstrated an acute MICT bout decreases (~10%–15%) (Ueda, Yoshikawa, Katsura, Usui, & Fujimoto, 2009; Ueda, Yoshikawa, Katsura, Usui, Nakao, & Fujimoto, 2009), increases (~18%–31%) (Laan et al., 2010; Martins et al., 2007; Shorten et al., 2009), or has no effect (~ − 3%–12%) (Balaguera‐Cortes et al., 2011; Erdmann et al., 2007; George & Morganstein, 2003; Hubert et al., 1998; Jokisch et al., 2012; Kelly et al., 2012; King et al., 1994, 1996; King, Wasse, Ewens, et al., 2011; Laan et al., 2010; Lage et al., 2010; Larson‐Meyer et al., 2012; Melby et al., 2002; O'Donoghue et al., 2010; Pomerleau et al., 2004; Shorten et al., 2009; Unick et al., 2010; Vatansever‐Ozen et al., 2011) on energy intake. Following the meta‐analysis (Schubert et al., 2013), results continue to remain equivocal demonstrating decreases (~25%–48%); (Beer et al., 2020; Islam et al., 2017; Sim et al., 2014) or no effect (~ − 2%–11%); (Alajmi et al., 2016; Deighton et al., 2013; Douglas et al., 2017; King et al., 2017; Martins et al., 2015; McCarthy et al., 2023; Panissa et al., 2016; Rossato & Fuchs, 2014; Sim et al., 2014; Ueda, Yoshikawa, Katsura, Usui, & Fujimoto, 2009), however of the studies that did not detect an effect, 4 measured relative energy intake and all detected a decline in relative energy intake by ~9%–15% (Alajmi et al., 2016; Deighton et al., 2013; Douglas et al., 2017; Panissa et al., 2016). While the majority of individual study results demonstrate no effect of MICT on subsequent energy intake, it is important to highlight issues (inaccuracies due to over‐ or underestimation of food intake) related to measuring energy intake (Fazzino et al., 2023; Gregersen et al., 2008; Rossato & Fuchs, 2014) and that most studies do not have sufficient sample sizes to detect meaningful changes. Additionally, the measurement of energy intake varies in method and timing with free‐living (Beer et al., 2020; Bornath et al., 2023; Islam et al., 2017; McCarthy et al., 2023; Moniz et al., 2023; Sim et al., 2014; Vanderheyden et al., 2020) or a postexercise ad‐libitum meal within immediately to 8 h postexercise (Balaguera‐Cortes et al., 2011; Douglas et al., 2017; Erdmann et al., 2007; George & Morganstein, 2003; Hubert et al., 1998; Jokisch et al., 2012; Kelly et al., 2012; King et al., 1994, 1996, 2017; King, Wasse, Ewens, et al., 2011; Laan et al., 2010; Lage et al., 2010; Larson‐Meyer et al., 2012; Melby et al., 2002; O'Donoghue et al., 2010; Pomerleau et al., 2004; Shorten et al., 2009; Ueda, Yoshikawa, Katsura, Usui, & Fujimoto, 2009; Unick et al., 2010; Vatansever‐Ozen et al., 2011).

### 2.2 Resistance exercise

Most research on exercise‐induced appetite suppression uses aerobic exercise, however the original meta‐analyses included resistance training (RT) studies (Balaguera‐Cortes et al., 2011; Ballard et al., 2009; Broom et al., 2009; Laan et al., 2010). Understanding the role of RT on appetite regulation is important as RT is crucial for muscle strength and other musculoskeletal, metabolic, and health benefits (Phillips et al., 2023). Studies assessing RT identify decreases in acylated (~45%–60%) (Balaguera‐Cortes et al., 2011; Broom et al., 2009; McCarthy et al., 2024) and total ghrelin (~27%–31%) (Halliday et al., 2021; Liu et al., 2023; Purcell et al., 2023), with one demonstrating no change in acylated ghrelin (~9%) (Larsen et al., 2017). PYY does not appear responsive to RT as most studies identify no changes (~3%–7%) in active (McCarthy et al., 2024) or total (Balaguera‐Cortes et al., 2011; Broom et al., 2009; Halliday et al., 2021; Rahmani‐Nia et al., 2015) and only two have shown increases (~10%) in active (Liu et al., 2023) or total (Purcell et al., 2023). Few studies have assessed the effect of RT on either active (Larsen et al., 2017; McCarthy et al., 2024) or total GLP‐1 (Halliday et al., 2021) all identifying no changes (7%–10%). Taken altogether, both acylated and total ghrelin appear affected by RT, with little influence on PYY or GLP‐1. The effect of RT on subjective appetite is unclear as studies demonstrate decreases by ~45% (Broom et al., 2009; McCarthy et al., 2024) or no change (2%–5%) (Cadieux et al., 2014; Halliday et al., 2021; Larsen et al., 2017; Purcell et al., 2023). There also appears to be no effect of RT on energy intake as all studies demonstrate no differences (~1%–9%) in ad‐libitum meal intake 15–120 min postexercise (Balaguera‐Cortes et al., 2011; Cadieux et al., 2014; Halliday et al., 2021; Purcell et al., 2023) or free‐living energy intake (McCarthy et al., 2024). Considering the vast health benefits of RT exercise (Phillips et al., 2023), more work should focus on understanding the appetite response to RT and use different RT protocols as many of the aforementioned studies (Balaguera‐Cortes et al., 2011; Broom et al., 2009; Cadieux et al., 2014; Halliday et al., 2021; Larsen et al., 2017) included RT as a comparator group to aerobic and have not focused solely on RT.

## 3 PROPOSED MECHANISMS

In 2016, eight mechanisms potentially involved in the exercise‐induced changes in appetite‐regulation were proposed (Hazell et al., 2016). Here we provide updates regarding lactate, IL‐6, temperature, and blood glucose, while the other mechanisms have received little attention in the past decade and future research remains warranted.

### 3.1 Lactate

Lactate has garnered the most attention and it's role has been recently reviewed (McCarthy et al., 2020). Causative evidence from rodent models demonstrates peripheral and central lactate administration (i.e., infusion or injection) alter both peripheral (Engelstoft et al., 2013) and central (Cha & Lane, 2009; Chen et al., 2023; Ou et al., 2019; Torres‐Fuentes et al., 2019) appetite pathways involved in reductions in energy intake (Cha & Lane, 2009; Chen et al., 2023; Lam et al., 2008; Langhans et al., 1985; Nagase et al., 1996; Silberbauer et al., 2000). Lactate has been infused in humans in two studies (increasing lactate ~1.2–2.5 mmol∙L^−1^; similar to increases following low‐intensity exercise) where one study demonstrated lower energy intake (~1046 kJ) from an ad libitum meal compared to a saline infusion when participants were in an euglycemic state (Schultes et al., 2012) and the other demonstrated reduced acylated ghrelin concentrations, but no concomitant change in hunger/satiety (Pedersen et al., 2022). Exercise studies using both aerobic (Islam et al., 2017; McCarthy et al., 2023; Sim et al., 2014; Vanderheyden et al., 2020) and RT (Freitas et al., 2020; Liu et al., 2023; McCarthy et al., 2024) demonstrate greater lactate accumulation (≥2–2.5 mmol∙L^−1^) aligns with suppressed subjective appetite perceptions and/or energy intake likely via changes in appetite‐regulating hormones. One of the most compelling studies in humans assessing lactate's role in appetite used a unique design where participants ingested sodium bicarbonate pre‐exercise allowing increased lactate accumulation during exercise and examined it's effects on appetite‐regulating parameters. The exercise sessions were identical so any differences between sessions could be attributed to greater lactate accumulation. Greater blood lactate accumulation (+2.7 mmol∙L^−1^) was achieved with sodium bicarbonate ingestion coinciding with ~30% lower acylated ghrelin concentrations and tended to reduce subjective appetite perceptions (~20%) compared to placebo (Vanderheyden et al., 2020). Recent evidence in rodent models (Lund et al., 2023) using a series of well‐designed experiments disputes lactate's potential role and suggests it is the high osmolarity of the injected/infused lactate solution that leads to the reductions in energy intake in rodent models, not the lactate itself. While this data (Lund et al., 2023) suggests the reductions in energy intake are due to the high osmolarity of the solutions causing malaise and not lactate, it does not account for the changes in peripheral or central appetite pathways that have been demonstrated in vitro and in vivo (Cha & Lane, 2009; Chen et al., 2023; Engelstoft et al., 2013; Ou et al., 2019; Torres‐Fuentes et al., 2019). Perhaps injecting/infusing lactate may not be the most effective method to study lactate's role in appetite regulation unless the osmolarity of the lactate and placebo solutions are matched. Additionally, the exogenous administration of lactate does not reflect that natural efflux of lactate from skeletal muscle. More causative work is required to fully elucidate lactate's role on appetite and confirm proposed mechanisms.

### 3.2 Interleukin‐6

IL‐6 has received attention as a potential mechanism for exercise‐induced appetite suppression as it's production and release is closely related to exercise intensity (Ostrowski et al., 2000; Pedersen & Fischer, 2007), duration (Ostrowski et al., 1998; Pedersen & Fischer, 2007), and the amount of muscle mass involved (Pedersen & Fischer, 2007). Early evidence supports a role in appetite regulation as previously reviewed (Ellingsgaard et al., 2011; Hazell et al., 2016; Kahles et al., 2014; Shirazi et al., 2013), but despite these promising results more recent work has been contradictory. Incubating GLP‐1 producing cells (GLUTag cells) with IL‐6 had no effect on GLP‐1 production and infusing IL‐6 into perfused mouse small intestine had no effect on GLP‐1 release (Christiansen et al., 2018). High doses of IL‐6 have been shown to suppress ghrelin mRNA and protein expression in pancreatic cell lines (Chew et al., 2014; Lao et al., 2013) though no work has followed up on this. In humans, IL‐6 was moderately correlated with GLP‐1 (no relationship with ghrelin) following acute bouts of MICT, VICT, and SIT in young normal weight males (Islam et al., 2017), though in young lean sedentary males as well as those experiencing obesity there was no effect of exercise‐induced or adiposity‐related IL‐6 on GLP‐1, acylated ghrelin, or other appetite markers (Bornath et al., 2023). Taken together, IL‐6's potential role in appetite regulation is still unclear with inconclusive results in vivo and in vitro. Additionally, IL‐6 release with exercise may be driven by lactate (Hojman et al., 2019) making it difficult to discern it's role.

### 3.3 Temperature

The role of temperature has garnered attention as a potential mechanism in appetite regulation in the past decade (Brobeck, 1948; Hazell et al., 2016), and a recent meta‐analysis revealed a modest orexigenic effect of cold exposure and a small anorexigenic effect of heat exposure (Millet et al., 2021). The exact mechanisms eliciting these responses are unclear and recent studies on environmental temperature at rest (Zakrzewski‐Fruer et al., 2021) or following exercise (Kojima et al., 2018; Laursen et al., 2017) demonstrate increased acylated ghrelin following exercise in cold temperatures but no effect on total GLP‐1 or total PYY (Mandic et al., 2019). Future work should look to explore the potential mechanisms involved in how changes in body/environmental temperature are involved in the appetite‐regulatory response.

### 3.4 Blood glucose and insulin

Changes in blood glucose and insulin have long been hypothesized to regulate appetite postprandially (Campfield & Smith, 2003; Mayer, 1955) as postprandial glycemic dips predict increases in appetite and energy intake (Wyatt et al., 2021) and insulin has been proposed to inhibit ghrelin secretion (Gagnon & Anini, 2012). While high blood glucose can attenuate ghrelin secretion from gastric mucosal cells in culture (Sakata et al., 2012) and intestinal glucose absorption stimulates GLP‐1 release from enteroendocrine cells (Lu et al., 2021), whether brief (≤30 min) increases in blood glucose and insulin follow higher‐intensity exercise (Peake et al., 2014; Vincent et al., 2004) are involved in exercise‐induced appetite suppression is unclear. Preliminary work from our group suggests increases in glucose postexercise do not contribute to exercise‐induced appetite suppression as despite similar increases in plasma glucose following MICT and SIT, acylated ghrelin and subjective appetite were suppressed following SIT only, though MICT increased GLP‐1 (Bornath et al., n.d.). Insulin decreased following the standardized meal with no differences between sessions (Bornath et al., n.d.). It is plausible that blood glucose and insulin are important regulators postprandially but systemic metabolic changes during exercise override their effects, though more work is necessary to fully elucidate their role.

The remaining proposed mechanisms (redistribution of blood flow, sympathetic nervous system activity, gastrointestinal motility, and free fatty acid concentrations) have garnered little attention despite being implicated as potential mechanisms for exercise‐induced appetite suppression. More work is needed to determine whether they are important and enhanced understanding of the potential mechanisms will be helpful in understanding the variability associated with exercise‐induced changes in appetite regulation (Hazell et al., 2016).

## 4 FUTURE DIRECTIONS

**FIGURE 3:**
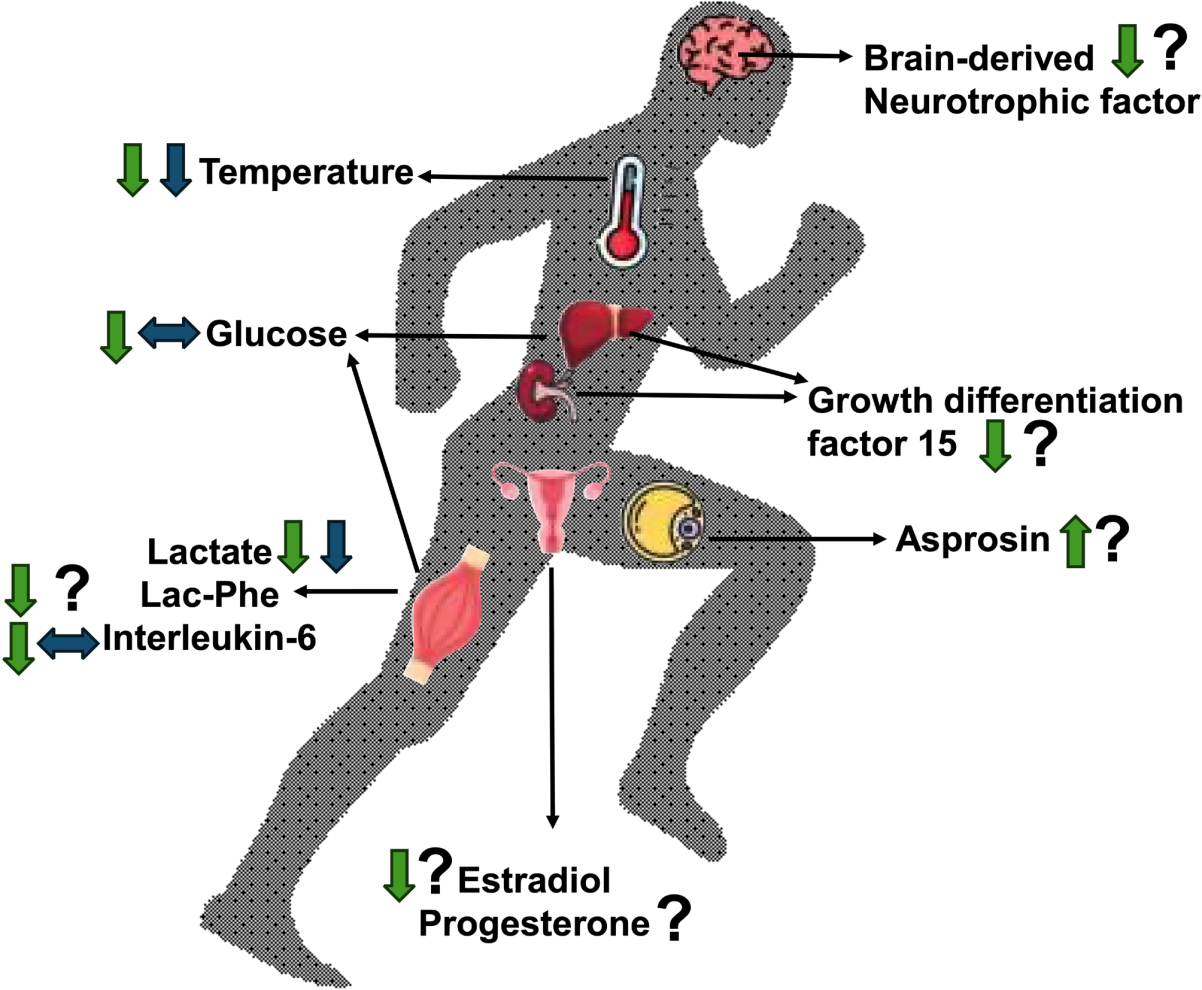
Overview of potential modulators involved in exercise‐induced appetite suppression. Green arrows represent the hypothesized effect on appetite. Blue arrows indicate that human data shows no effect (sideways) or an effect (downward) and a question mark indicates data is unclear or lacking.

There are additional potential modulators that may be involved in exercise‐induced appetite suppression including sex hormones, recently identified appetite hormones, as well as “myokines” and “exerkines” (Figure 3).

### 4.1 Sex hormones

A majority of research (~85%) on exercise and appetite included only males as the menstrual cycle has been viewed as a confounding factor as opposed to a relevant physiological modulator of appetite. It has been suggested that estradiol (E_2_) (Butera, 2010; Sinchak & Wagner, 2012) may be appetite‐inhibiting while progesterone is appetite‐stimulating solely in the presence of E_2_ (Butera, 2010; Hirschberg, 2012) and this has been supported by research demonstrating energy intake increases during the LP compared to the FP (Gorczyca et al., 2016; Hirschberg, 2012; Tucker et al., 2024). Only two studies have compared exercise‐induced appetite suppression in premenopausal females in the FP and LP. The first showed no differences in acylated ghrelin, total PYY, appetite perceptions, or energy intake following an acute bout of MICT (Kamemoto et al., 2022). The second study demonstrated a blunted acylated ghrelin response in the LP following an acute bout of VICT with no differences in active PYY, active GLP‐1, or appetite perceptions, though there was increased energy intake in the LP (Moniz et al., 2023). These differences may be due to methodological differences, as participants in the first study arrived at the laboratory fasted and were not fed for several hours following exercise resulting in ~16 h without food (Kamemoto et al., 2022), whereas in the latter participants were fed pre‐exercise upon arrival at the laboratory (Moniz et al., 2023). Taken together, it is possible that the menstrual cycle may influence this response, though there is limited research investigating the influence of ovarian hormones on the appetite‐specific response to exercise and more work is warranted.

### 4.2 Growth differentiation factor 15

Growth differentiation factor 15 (GDF15) is a stress response cytokine (Tsai et al., 2018) released from a variety of tissues (i.e., liver, kidney, lung, intestines, and placenta) where pharmacological doses can suppress energy intake and reduce body weight (Emmerson et al., 2017; Hsu et al., 2017; Mullican et al., 2017; Yang et al., 2017). A potential role in exercise‐induced appetite suppression has been suggested as prolonged exercise (>2 h in duration at moderate‐vigorous intensities) increases circulating concentrations (~400%–500%) similar to those associated with pathological conditions or attained following metformin treatment, though this response is lost when exercise duration is shorter (≤1 h at moderate‐vigorous intensities) (Klein et al., 2021, 2022; Kleinert et al., 2018). GDF15's potential role in appetite was recently reviewed (Klein et al., 2022) and it is unclear whether it contributes to exercise‐induced appetite suppression as changes in GDF15 postexercise have yet to demonstrate reductions in food intake (Klein et al., 2021). While supraphysiological endogenous doses of GDF15 can reduce food intake and body weight, more work is required to determine if it is involved in exercise‐induced appetite suppression.

### 4.3 Lac‐Phe

A recent advancement related to lactate's potential role in appetite regulation is linked to a lactate derived metabolite N‐lactoyl‐phenylalanine (Lac‐Phe), which has been shown to be one of the most highly circulating metabolites following exercise in mice and humans (Li et al., 2022). Acute Lac‐Phe injection reduces food intake in diet‐induced obese mice by ~50% and daily injections reduce body weight by ~7% over a 10‐day period (Li et al., 2022). This dose was ~100‐fold greater than circulating concentrations following exercise (Lund et al., 2022) questioning whether physiological doses have appetite‐suppressing effects. Acylated ghrelin concentrations 30 min following Lac‐Phe injection were ~ 50% lower compared to a saline injection, though the sample size was only 3 mice, and no *p*‐value was provided. Though it appears Lac‐Phe may affect acylated ghrelin concentrations, a mechanism has yet to be established as to how Lac‐Phe suppresses appetite (Lund et al., 2022), thus more work is needed to determine whether physiological concentrations of Lac‐Phe produced during acute exercise are capable of suppressing appetite and the potential mechanisms involved.

### 4.4 Brain‐derived neurotrophic factor

Brain‐derived neurotrophic factor (BDNF) is a neurotrophin expressed in several key areas of the brain that plays a key role in synaptic plasticity as well as neuronal survival and function (Patapoutian & Reichardt, 2001; Poo, 2001). It has been implicated in appetite (Rios, 2013) as central administration reduces food intake (~94%) and body weight (~32%) in rodents (Pelleymounter et al., 1995), and BDNF knockout mice develop hyperphagia and obesity (Fox et al., 2013). While peripheral concentrations of BDNF increase postexercise (~13%–190%) on an intensity‐dependent basis in humans (Ceylan et al., 2023; Marston et al., 2017; Rasmussen et al., 2009; Reycraft et al., 2020), no study has explored it's relevance to appetite in humans by assessing subjective appetite perceptions or energy intake (ad libitum or free‐living) in conjunction with BDNF. It appears BDNF's effects on appetite occur solely in the brain (Rios, 2013) making it difficult to assess in humans, though future work should attempt to use advanced techniques to determine if peripheral increases in BDNF contribute to exercise‐induced appetite suppression in humans.

### 4.5 Asprosin

Asprosin is a recently discovered fasting‐induced glycogenic hormone released from white adipose tissue (Romere et al., 2016). Adults experiencing obesity and obese mice have greater concentrations of fasting asprosin compared to their lean counterparts (Ceylan et al., 2023; Duerrschmid et al., 2017; Romere et al., 2016) and subcutaneous administration of asprosin to mice increases food intake via activation of AgRP neurons and inhibition of POMC neurons that were prevented with ablation of AgRP neurons (Duerrschmid et al., 2017). Exercise (MICT and HIIT) appears to reduce asprosin concentrations (~20%–24%) in both normal weight and individuals living with obesity independent of intensity (Ceylan et al., 2020, 2023) though a single supramaximal sprint found no change in males but a small increase in females (Wiecek et al., 2018). None of the studies that have assessed changes in asprosin have measured other outcomes related to appetite (i.e., appetite perceptions or energy intake) making it difficult to discern asprosin's role in exercise‐induced appetite suppression in humans.

## 5 CONCLUSION

The field of exercise and appetite‐regulation is still growing and most evidence suggests exercise‐induced appetite suppression via changes in peripheral appetite‐regulating hormones, mostly acylated ghrelin, and potential reductions in subsequent energy intake. Small samples sizes (*n* = 8–12) are an issue as most studies are powered to detect changes in peripheral appetite‐regulating hormones but not subjective measures of appetite perceptions or energy intake. It is important that this field continues to progress using rigorous approaches such as sample size calculations, clinical trial registration (where applicable, see (Richter et al., 2024) for a commentary on this), and sex‐based comparisons to answer important questions. The next steps in exercise and appetite‐regulation should continue to focus on the mechanisms responsible for the changes and consider the modulating effects of sex hormones, and other myokines/exerkines in studies aimed at improving our understanding of how exercise can suppress appetite.

## AUTHOR CONTRIBUTIONS

All authors were involved in all aspects of this review.

## FUNDING INFORMATION

No funding was required to directly support this review. However, much of the work cited from our group was supported by grants from the Candadian Natural Sciences and Engineering Research Council (NSERC; RGPIN 2016‐ 06118 & RGPIN 2022‐03991).

## CONFLICT OF INTEREST STATEMENT

All authors have reviewed and approved the final manuscript. We declare no conflicts of interest that could potentially influence the content or conclusions presented in this review.

## ETHICS STATEMENT

This manuscript is a review article and does not involve any original data collection using human participants or animals. Therefore, no ethics approval was required.

## Data Availability

All data presented is sourced from published academic literature. A complete list of references is provided.
